# Prioritization of antiretroviral therapy in patients with high CD4 counts, and retention in care: lessons from the START and Temprano trials

**DOI:** 10.1002/jia2.25077

**Published:** 2018-02-13

**Authors:** Mauro Schechter

**Affiliations:** ^1^ Projeto Praça Onze Universidade Federal do Rio de Janeiro Rio de Janeiro Brazil

**Keywords:** HIV, AIDS, Antiretroviral therapy, health systems, retention in care, policy

## Abstract

Initiation of antiretroviral therapy is not a once in a lifetime opportunity. In some resource constrained settings financial limitations make it necessary to prioritize treatment initiation for some groups of patients. In developed countries, there are patients who are reluctant to initiate treatment. Subgroup analysis of the START trial can inform recommendations for which patients with CD4 counts >500 cells mm^3^ temporary postponement of treatment initiation is safer. These include individuals aged <30 years and/or with CD4/CD8 ratio of >0.8 and/or viral load of <5000. This is because these individuals are at very low risk of disease progression in the subsequent 2 to 3 years, the risk is minimally diminished by antiretroviral therapy and is virtually identical in the first 18 months of therapy regardless of treatment initiation. In addition, asymptomatic young individuals are at higher risk of loss‐to‐follow and of low adherence to treatment, and those with low viral loads are less likely to transmit the virus. In addition, lessons from START and Temprano can help design trials to investigate strategies to decrease losses‐to‐follow‐up, while minimizing risks to patients.

In countries in resource limited settings (RLS) that cannot afford to treat all HIV‐infected individuals, the World Health Organization (WHO) recommends prioritizing the treatment of patients with CD4 counts of <350 cells/mm^3^. For less resource constrained settings that can afford to treat some but not all patients with CD4 counts of >500 cells/mm^3^, there are no specific recommendations 1. Almost all guidelines, particularly from developed countries, stress the importance of patient readiness for starting therapy 2. Unfortunately, there are very few specific recommendations to help health care providers persuade reluctant patients with high CD4 counts to start therapy.

Considering the need for prioritization in certain RLS for patients with CD4 counts >350 cells/mm^3^ and for respecting patient readiness in developed and developing countries alike, here we attempt to discuss how data from the START 3 trial can help inform decisions. We also discuss how data from the START and Temprano 4 trials might help designing trials to evaluate strategies to improve retention in care.

The optimal CD4 cell count at which ART should be initiated in asymptomatic individuals has been a hotly debated topic for three decades 5. In 2015, all major international treatment guidelines were updated to incorporate the results of the START and Temprano trials.

START was a large international trial that involved 4685 previously untreated asymptomatic adult HIV‐positive persons from 35 countries in five continents with CD4 counts of >500 cells/mm^3^. Over 50% of the patients were from RLS. Participants were randomized to immediate initiation of ART or its deferral until the CD4 count declined to <350 cells/mm^3^. After a mean follow‐up of 3 years, the primary endpoint (death, serious AIDS or serious non‐AIDS event) had occurred in 1.8% and 4.1% of the participants in the immediate and in the deferred groups respectively. There was no evidence that the beneficial effect of immediate ART varied among various predefined subgroups, including region of the world. These results were interpreted as indicative that ART should be initiated in all asymptomatic HIV‐positive adults regardless of CD4^+^ count. Furthermore support for this interpretation was provided by the Temprano trial, which was conducted in Ivory Coast and included 2076 asymptomatic patients with CD4 counts of <800 cells/mm^3^. Patients were randomized to immediate ART or deferral until reaching contemporary WHO guidelines (CD4 < 200 and later <350 cells/mm^3^). When the analysis was restricted to participants with CD4 counts of >500 cells/mm^3^ at study entry (n = 868), the cumulative probability of death or severe HIV‐related illness (the primary endpoint) at 30 months declined from 12.4% to 6.9% in the deferred and immediate arms respectively.

Although in the START trial the relative risk reduction for the primary endpoint was large for all subgroups, absolute differences varied considerably. In some subgroups, the absolute risk of disease progression was substantial and thus the absolute impact of ART was numerically sizable. For example, for those over 50 years of age at study entry, 11.7% in the deferred arm had reached an endpoint by 36 months, compared to 2.9% in the immediate arm, a difference of 8.8%. In this subgroup, it would be necessary to treat 45 individuals to prevent one event. At the other extreme, three characteristics were associated with a low risk of progression during the follow up period, namely age of <30 years, CD4/CD8 ratio of >0.8, and viral load of <3000 (or 5000) copies/mm^3^ at study entry. For example, for those with a viral load of <3000 copies/mm^3^ (25% of all study participants) the absolute risks of reaching an endpoint at 3 years of follow‐up were 2.4 and 1.9% in the immediate and deferred groups respectively, a difference of only 0.5%. In this subgroup, it would be necessary to treat 992 individuals to prevent one event. Moreover, the two curves in this subgroup were completely superimposable in the first 2 years of follow‐up 6. Furthermore, of the 76 participants in the deferred group who had the three characteristics associated with a lower risk of reaching an endpoint (age of <30, viral load of <5000, and CD4/CD8 ratio of >0.8) only one experienced an endpoint event after 3.2 years of follow‐up (Birgit Grund, personal communication).

In START participants were censored on the date an endpoint was reached or they were last known to be alive. In addition, over 98% of the participants who started ART had an undetectable viral load at 12 months of follow‐up. Thus, results in START should represent the best obtainable benefit with present‐day ART. Participants in clinical trials have greater adherence and health seeking behaviours than the general population and many programs report losses to follow‐up greater than 50% in the first 2 years after treatment initiation. Thus, it seems fair to predict that the actual effectiveness of ART for individuals with high CD4 counts might be significantly inferior to the efficacy reported in START. Furthermore, immediate ART might be even less effective in subgroups which have even higher rates of loss‐to‐follow up, such as healthy young individuals with high CD4 counts, which had the smallest absolute benefit in START. For these individuals, the future advent of long acting drugs, which might circumvent adherence concerns, is likely to affect the balance.

The term “immediate” can be misleading. In the START trial, the reported median time since HIV diagnosis to being referred to the study was 1 year. The median time since the first study CD4 cell count and ART initiation in the immediate arm was 6 weeks. Hence, for most individuals in the immediate arm of START over 1 year had elapsed between diagnosis and ART initiation. On the other hand, “immediate” is often misinterpreted as referring to something that occurs suddenly. Thus, the concept of “immediate” utilized in START for treatment initiation is substantially different from its colloquial use or its meaning in the proposed strategy of same‐day HIV testing and ART initiation.

The strategy of whether same‐day HIV testing and ART initiation improves retention in care and virologic suppression was tested in a randomized trial conducted in Haiti involving 564 individuals with CD4 cell counts of >500 cells/mm^3^. The number of participants retained in care and with a 12‐month viral load of <50 copies/mm^3^ were 120 (42%) and 151 (54%) in the standard ART and same‐day groups respectively. Although same‐day initiation represented a statistically significant improvement, the result obtained at 12 months was far from the lifelong 90% UNAIDS target 7.

The probability of sexual transmission of HIV is determined by behaviour and biologic factors. Results of various studies have demonstrated that infected individuals with suppressed viremia are unlikely to transmit the virus, adding an important societal benefit to ART 8, 9. Arguing against this unquestionable benefit of ART is the possibility of transmission of resistant viruses by individuals who do not adhere adequately to therapy.

The effect of the universal test and treat strategy as recommended by WHO on HIV acquisition at the population level was tested in a cluster randomized trial involving 22 communities in South Africa. In this trial, the universal test and treat strategy failed to lower HIV incidence. Thus, when postponement of treatment is considered, it might be reasonable to weigh the societal risks and benefits of providing ART to individuals who are at a lower risk of transmission of the virus and of disease progression in the short term, but who are at high risk of low adherence. For example, asymptomatic young persons with high CD4 counts^9^ and low viral loads 10, who are at low risk of disease progression and high risk of low adherence and thus of transmission of resistant viruses.

The credibility of subgroup analysis to provide recommendations has been questioned 11. In the case of the START trial, in addition to the very large sample size of most of its subgroups, several measures were taken to increase the reliability of its findings. These included defining subgroups at baseline and assessing heterogeneity of absolute risk reduction across subgroups using bootstrap tests.

The Temprano trial used a 2 × 2 factorial design in which participants were randomized to immediate or deferred ART according to contemporary WHO criteria, each with or without a 6‐month course of primary tuberculosis prophylaxis with isoniazid (IPT). Final results demonstrated that both in the entire study population and in the group with CD4 counts of >500 cells/mm^3^ at study entry, IPT had an independent and statistically significant positive impact on severe morbidity, the main outcome of the study.

Initiation of ART is not a once in a lifetime opportunity. As mentioned above, in some subgroups, including young individuals with high CD4 counts, low viral load, and preserved CD4/CD8 ratio, the risk of progression and the impact of ART over the ensuing one to 1 years are both low. Thus, a testable hypothesis to decrease losses‐to‐ follow‐up, while at the same time minimizing risks to patients, would be to combine lessons from START and Temprano to design studies of same‐day initiation, shown to improve outcomes as mentioned before. Inclusion could be restricted to individuals with CD4 counts >500 cells/mm^3^ with characteristics that predict low probability of disease progression but high probability of loss‐to‐follow‐up (e.g. young age and high CD4/CD8 ratio) 9. Randomization could then include deferral of ART initiation to arms providing IPT with and without financial incentives, to determine if retention in care would be improved.

In summary, in some RLS financial constraints make it necessary to prioritize treatment initiation for some groups of patients. In developed countries, there are patients who are reluctant to initiate ART. Subgroup analysis of the START trial can inform recommendations for which patients with CD4 counts >500 cells mm^3^ temporary postponement of treatment initiation is safer. These include individuals aged <30 years and/or with CD4/CD8 ratio of >0.8 and/or viral load of <5000. This is because these individuals are at very low risk of progression in the subsequent 2 to 3 years, the risk is minimally diminished by ART and is virtually identical in the first 18 months of therapy regardless of treatment initiation. In addition, asymptomatic young individuals are at higher risk of loss‐to‐follow and of low adherence to treatment 9, and those with low viral loads are less likely to transmit the virus 11.

Despite undisputable evidence of personal and societal benefits of ART, these benefits are small for some groups of individuals and little affected by ART in the short term. It has been demonstrated that biomarkers of inflammation and/or coagulation activation predict risk for serious cardiovascular disease, cancer, and all‐cause mortality 12. In several parts of the world, financial and infrastructure constraints mandate that prioritization remain necessary. In other settings, where resources are less constrained, individualized care will remain the norm. In these situations, validated algorithms that include simple demographic data, CD4/CD8 ratio, viral load, and biomarkers of inflammation and of coagulation activation may help further refine recommendations on how to assist on more accurately defining patients with CD4 counts of >500 cells/mm^3^ with the greatest need for immediate antiretroviral therapy.

## Competing interests

Participation in advisory boards and/or honoraria for lectures – Gilead Sciences, GSK/ViiV, Janssen, Merck.
